# Supplementary material for: High prevalence of Zika virus infection in populations of Aedes aegypti from South-western Ecuador
Source: PLoS Negl Trop Dis. 2024 Jan 18;18(1):e0011908. doi: 10.1371/journal.pntd.0011908 (PMC10826935; doi:10.1371/journal.pntd.0011908)
Supplement: S1 Table — (DOCX) [file pntd.0011908.s001.docx]

Supplementary Table S1. Average mosquito pool size for each sex, region and year.

| **City** | **Year** | **Mean pool size (SD)** | |
| --- | --- | --- | --- |
|  |  | female | male |
| Huaquillas | 2016 | 1.9 (2.0) | 2.3 (2.4) |
|  | 2017 | 3.1 (2.7) | 3.2 (2.6) |
|  | 2018 | 2.1 (1.8) | 2.1 (2.0) |
| Machala | 2016 | 1.8 (1.7) | 2.5 (2.4) |
|  | 2017 | 2.4 (1.9) | 3.1 (2.9) |
|  | 2018 | 1.6 (1.1) | 1.9 (1.7) |
| Portovelo | 2016 | 1.3 (0.6) | 1.3 (0.6) |
|  | 2017 | 2.0 (1.7) | 2.2 (1.8) |
|  | 2018 | 2.2 (2.0) | 2.1 (2.2) |
| Zaruma | 2016 | 1.5 (1.1) | 1.7 (1.5) |
|  | 2017 | 2.6 (2.3) | 2.5 (2.2) |
|  | 2018 | 2.2 (2.1) | 1.9 (2.0) |
