# Supplementary material for: High prevalence of Zika virus infection in populations of Aedes aegypti from South-western Ecuador
Source: PLoS Negl Trop Dis. 2024 Jan 18;18(1):e0011908. doi: 10.1371/journal.pntd.0011908 (PMC10826935; doi:10.1371/journal.pntd.0011908)
Supplement: Attachment — Submitted filename: Lopez-Rosero et al. - review.docx [file pntd.0011908.s002.docx]

High prevalence of Zika virus infection in populations of *Aedes aegypti* from South-western Ecuador.

Lopez-Rosero et al.

This is a short report describing the prevalence of Zika virus infection among the *Aedes aegypti* mosquitoes from four cities of El Oro province in Ecuador. The intriguing question arises: why has the Zika virus suddenly disappeared in recent years? To address this issue the authors conducted an investigation into the prevalence of Zika virus infections in mosquitoes, encompassing the peak transmission season, as well as the periods preceding and following it.

They observed highest infection rates among mosquitoes collected during post-peak transmission season. Notably, male mosquitoes displayed significant infections, indicating evidence of vertical transmission. The authors suggested that the virus is maintained within the mosquito population via vertical transmission, particularly in the absence of reported human cases. However, authors did not delve into whether the mosquitoes harbored any infectious virus particles. The potential contribution of venereal transmission is completely ignored. The authors do not offer any insights into why mosquitoes from the post-rainy season exhibited more infections compared to those collected during the peak transmission season. A sero survey might shed light on whether asymptomatic infections or immunity resulting from natural infections have contributed to the drastic reduction in human cases. The authors should also note that several infected mosquitoes in different pools could originate from the same infected parent.

Few minor comments:

what is the average number of mosquitoes per pool?

Table 3, indicate reference number under primer source
