# Supplementary material for: High prevalence of Zika virus infection in populations of Aedes aegypti from South-western Ecuador
Source: PLoS Negl Trop Dis. 2024 Jan 18;18(1):e0011908. doi: 10.1371/journal.pntd.0011908 (PMC10826935; doi:10.1371/journal.pntd.0011908)
Supplement: Attachment — Submitted filename: Lopez-Rosero et al_2023_response to reviewers_4.docx [file pntd.0011908.s003.docx]

Nicosia, October 25^th^, 2023

Dr. Mariangela Bonizzoni

Dr. Andrea Marzi

Editors,

PLoS Neglected Tropical Diseases

Dear Editors,

On behalf of the co-authors of the article entitled ‘High prevalence of Zika virus infection in populations of *Aedes aegypti* from South-western Ecuador’, I would like to thank the anonymous reviewers for their insightful comments and suggestions, which have undoubtedly helped improve the quality of this work.

It is my pleasure to submit a revised version of our manuscript, where we have addressed all the comments and request made by the reviewers. Below, we present a detailed description of said comments, and the way we have addressed them. Please notice that reviewer’s comments are presented in black font and between quotation marks, while author’s responses are presented in blue font. All line numbers mentioned correspond to those on the file named ‘Lopez et al-2023-PLoS NTD-revised_4-clean’ (version without tracked changes).

**Detailed responses to comments:**

“The authors did not delve into whether the mosquitoes harbored any infectious virus particles.”

On the updated version, we address this point in the discussion section (lines 360-367).

“The potential contribution of venereal transmission is completely ignored.”

On the updated version, we now discuss the potential roles of venereal transmission (lines 317-331, 358-359).

“The authors do not offer any insights into why mosquitoes from the post-rainy season exhibited more infections compared to those collected during the peak transmission season.”

On the updated version, we address this point in the discussion section (lines 355-359).

“A sero survey might shed light on whether asymptomatic infections or immunity resulting from natural infections have contributed to the drastic reduction in human cases.”

Although performing a sero-survey on the human population was not within the scope of our work (which is mainly entomological), we agree that in the future, performing this kind of research could help establishing the contribution of herd immunity to the epidemiological landscape of ZIKV on this region. On the updated version, we address this point in the discussion section (lines 368-377, 382-385).

“The authors should also note that several infected mosquitoes in different pools could originate from the same infected parent.”

Done. We have included this information in the discussion section (lines 340-342).

“What is the average number of mosquitoes per pool?”

On the updated version, this data is included in supplementary table S1, and a reference to this table is included in the results section (line 251).

“Table 3, indicate reference number under primer source”

Done. On the updated version, reference numbers are included in Table 3.

In addition to these improvements, and at the request of your editors, we have re-drawn Figure 1 using only data sources that are in the public domain, and therefore compatible with PLoS’ CC BY 4.0 license (lines 515-517; acknowledgement regarding the former figure was eliminated on lines 392-393). Furthermore, we have also performed a few other minor adjustments to the discussion (lines 293, 309, 311-315, 332-334, 337-340, 341-355, 378-382, 387-388 and 525) and abstract sections (lines 59-63, 78-79), to improve readability and completeness of our manuscript. The details of all changes can be visualized in the file named ‘Lopez et al-2023-PLoS NTD-revised_4-tracked’, where the ‘track changes’ option is activated.

We would like to thank the reviewers and editors once again for their invaluable input, and hope that they find this revised version suitable for publication on this prestigious journal.

Best regards,

Marco Neira, PhD

Associate Research Scientist

Climate and Atmosphere Research Center (CARE-C) / EMME-CCI

Room 117, Fresnel Building

The Cyprus Institute

20 Konstantinou Kavafi Street, 2121, Nicosia, Cyprus

Tel: (+357) 22397510 | Email: m.neira@cyi.ac.cy
