# Supplementary material for: High prevalence of Zika virus infection in populations of Aedes aegypti from South-western Ecuador
Source: PLoS Negl Trop Dis. 2024 Jan 18;18(1):e0011908. doi: 10.1371/journal.pntd.0011908 (PMC10826935; doi:10.1371/journal.pntd.0011908)
Supplement: Attachment — Submitted filename: Lopez et al_2024-response to reviewers_6.docx [file pntd.0011908.s004.docx]

Nicosia, January 4^th^, 2024

Dr. Mariangela Bonizzoni

Dr. Andrea Marzi

Editors,

PLoS Neglected Tropical Diseases

Dear Editors,

On behalf of the co-authors of the article entitled ‘High prevalence of Zika virus infection in populations of *Aedes aegypti* from South-western Ecuador’, I would like to thank the anonymous reviewers for their insightful comments and suggestions, which have undoubtedly helped improve the quality of this work.

It is my pleasure to submit a revised version of our manuscript, where we have addressed the reviewers’ comments, as described below. Please notice that reviewers’ comments are presented in black font and between quotation marks, while our responses are presented in blue font. All line numbers mentioned correspond to those on the file named ‘Lopez et al-2024-PLoS NTD-revised_6-clean’ (version without tracked changes).

We would like to thank the reviewers and editors once again for their invaluable input, and hope that they find this revised version suitable for publication on this prestigious journal.

**--------------------------------------------------**

**Detailed responses to comments:**

“They have addressed my concerns. However, I would like to inform the authors that the genus Flavivirus has been renamed as Orthoflavivirus”

Done. We have changed the name of the genus to Orthoflavivirus throughout the text (lines 102, 215, 301).

Additionally, we have addressed the following technical check request, sent to us on a separate email on Jan 4, 2024:

“Please be so kind and specifically state whether written informed consent was OBTAINED from all adult participants or not”

Done. We have changed the wording of our ethics statement (lines 246-247) to clearly state that written informed consent was obtained.

--------------------------------------------------

Best regards,

Marco Neira, PhD

Associate Research Scientist

Climate and Atmosphere Research Center (CARE-C) / EMME-CCI

Room 117, Fresnel Building

The Cyprus Institute

20 Konstantinou Kavafi Street, 2121, Nicosia, Cyprus

Tel: (+357) 22397510 | Email: m.neira@cyi.ac.cy
